# Marburgvirus Resurgence in Kitaka Mine Bat Population after Extermination Attempts, Uganda

**DOI:** 10.3201/eid2010.140696

**Published:** 2014-10

**Authors:** Brian R. Amman, Luke Nyakarahuka, Anita K. McElroy, Kimberly A. Dodd, Tara K. Sealy, Amy J. Schuh, Trevor R. Shoemaker, Stephen Balinandi, Patrick Atimnedi, Winyi Kaboyo, Stuart T. Nichol, Jonathan S. Towner

**Affiliations:** Centers for Disease Control and Prevention, Atlanta, Georgia, USA (B.R. Amman, A.K. McElroy, K.A. Dodd, T.K. Sealy, A.J. Schuh, S.T. Nichol, J.S. Towner);; Uganda Virus Research Institute, Entebbe, Uganda (L. Nyakarahuka);; Emory University, Atlanta (A.K. McElroy);; University of California, Davis, California, USA (K.A. Dodd);; Centers for Disease Control and Prevention, Entebbe (T.R. Shoemaker, S. Balinandi);; Uganda Wildlife Authority, Kampala, Uganda (P. Atimnedi);; Uganda Ministry of Health, Kampala (W. Kaboyo)

**Keywords:** Marburgvirus, Rousettus aegyptiacus, bats, mine, miners, outbreak, disease ecology, viruses, Uganda, Marburg virus, Ravn virus, marburgviruses, extermination, depopulation

**To the Editor:** Marburg virus (MARV) and Ravn virus (RAVV), collectively called marburgviruses, cause Marburg hemorrhagic fever (MHF) in humans. In July 2007, 4 cases of MHF (1 fatal) occurred in miners at Kitaka Mine in southern Uganda. Later, MHF occurred in 2 tourists who visited Python Cave, ≈50 km from Kitaka Mine. One of the tourists was from the United States (December 2007) and 1 was from the Netherlands (July 2008); 1 case was fatal (*1*,*2*,*3*). The cave and the mine each contained 40,000–100,000 *Rousettus aegyptiacus* bats (Egyptian fruit bats).

Longitudinal investigations of the outbreaks at both locations were initiated by the Viral Special Pathogens Branch of the Centers for Disease Control and Prevention (CDC, Atlanta, GA, USA, and Entebbe, Uganda) in collaboration with the Uganda Wildlife Authority (UWA) and the Uganda Virus Research Institute (UVRI). During these studies, genetically diverse MARVs and RAVVs were isolated directly from bat tissues, and infection levels of the 2 viruses were found to increase in juvenile bats on a predictable bi-annual basis (*4*,*5*). However, investigations at Kitaka Mine were stopped when the miners exterminated the bat colony by restricting egress from the cave with papyrus reed barriers and then entangling the bats in fishing nets draped over the exits. The trapping continued for weeks, and the entrances were then sealed with sticks and plastic. These depopulation efforts were documented by researchers from UVRI, the CDC, the National Institute of Communicable Diseases (Sandringham, South Africa), and UWA during site visits to Kitaka Mine (Technical Appendix Figure). In August 2008, thousands of dead bats were found piled in the forest, and by November 2008, there was no evidence of bats living in the mine; whether 100% extermination was achieved is unknown. CDC, UVRI, and UWA recommended against extermination, believing that any results would be temporary and that such efforts could exacerbate the problem if bat exclusion methods were not complete and permanent (*6*,*7*).

In October 2012, the most recent known marburgvirus outbreak was detected in Ibanda, a town in southwest Uganda. Ibanda is ≈20 km from the Kitaka Mine and is the urban center that serves smaller communities in the Kitaka area. This MHF outbreak was the largest in Ugandan history: 15 laboratory-confirmed cases occurred (*8*). In November 2012, an ecologic investigation of the greater Ibanda/Kitaka area was initiated. The investigation included interviews with local authorities to locate all known *R. aegyptiacus* colonies in the area. Although minor colonies of small insectivorous bats were found, the only identifiable colony of *R. aegyptiacus* bats was found inside the re-opened Kitaka Mine, albeit at much reduced size, perhaps 1%–5% of that found before depopulation efforts.

To determine whether the *R. aegyptiacus* bats that had repopulated Kitaka Mine were actively infected with marburgviruses, we tested 400 bats by using previously described methods (*4*,*5*). Viral RNA was extracted from ≈100 mg of liver and spleen tissue by using the MagMAX Total Nucleic Acid Isolation Kit (Applied Biosystems, Foster City, CA, USA) according to the manufacturer’s recommended protocol. The Fisher exact test was conducted by using IBM SPSS Statistics, version 19.0 (IBM Corp., Armonk, NY, USA).

Of the 400 *R. aegyptiacus* bats collected, 53 (13.3%) were positive for marburgvirus RNA by quantitative reverse transcription PCR (32/233 [13.7%] adults and 21/167 [12.6%] juveniles; Technical Appendix Table); marburgvirus was isolated from tissue samples from 9 of the 400 bats. The overall level of active infection was significantly higher than that found in Kitaka Mine during 2007–2008 (5.1%) (*5*) (Fisher exact test, p<0.001) and in other studies in Uganda (Python Cave [2.5%]) and Gabon (4.8%) (*4*,*9*). The reason for the increase is not clear, but it may be related to the effects of the extermination and subsequent repopulation. Increases in disease prevalence in wildlife populations after culling are not unprecedented (*6*,*7*). We speculate that after the depopulation attempt, a pool of susceptible bats became established over time and was subjected to multiple marburgvirus introductions, as evidenced by the genetic diversity of viruses isolated from the bats (Figure). A pool of susceptible bats would have led to higher levels of active infection within the colony, thereby increasing the potential for virus spillover into the human population. A significant sex and age bias was not detected with respect to active infection during the breeding season (Fisher exact test, p>0.5 for both), and overall, the presence of virus-specific IgG among the bats was 16.5%, a finding consistent with that in previous studies (*4*,*5*).

**Figure:**
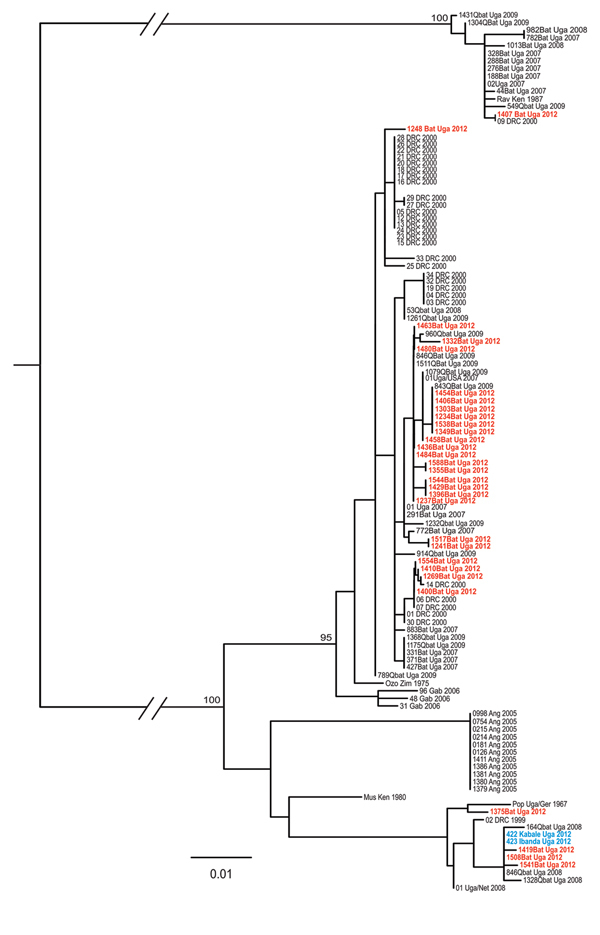
Phylogeny of concatenated marburgvirus nucleoprotein (NP) and viral protein 35 (VP35) gene fragments as determined by using the maximum-likelihood method. Sequences from the NP (289–372 nt) and VP35 (203–213 nt) genes were amplified and determined from viral RNA and then sequenced as described elsewhere (*4*). Sequence names shown in red font represent those generated from samples collected from bats during the November 2012 outbreak investigation at Kitaka Mine, Uganda. Sequence names in blue font represent those generated from samples obtained from marburgvirus-infected persons in Kabale and Ibanda, Uganda, in 2012. Multiple sequence alignments were generated, and a maximum-likelihood analysis was conducted on concatenated NP and VP35 (208–580 nt) sequences by using the PhyML method in conjunction with the GTR+I+G nucleotide substitution model implemented in SeaView version 4.2.12 (*10*). NP and VP35 gene sequences determined from samples in this study (in red) were submitted to GenBank (accession nos. KJ747211–KJ747234 and KJ747235–KJ747253, respectively). Bayesian posterior probabilities above 50 are shown at the nodes. Scale bar indicates nucleotide substitutions per site. Ang, Angola; DRC, Democratic Republic of Congo; Gab, Gabon; Ger, Germany; Ken, Kenya; Net, Netherlands; Rav, Ravn virus; Uga, Uganda; Zim, Zimbabwe.

Phylogenetic analysis of viral RNA genome fragment sequences in this study showed high marburgvirus genetic diversity, including the presence of RAVVs and MARVs. Sequences for isolates from 3 bats were nearly identical to those of the MARV isolates obtained from patients in the 2012 Ibanda outbreak (*8*), suggesting that bats from Kitaka Mine were a likely source of the virus.

Technical Appendix Photographs taken during August 2008–September 2009 of bat extermination efforts at Kitaka Mine, and table showing demographic characteristics of bats captured during a Marburg hemorrhagic fever outbreak investigation at the mine in November 2012, Uganda.
