# Supplementary material for: Marburgvirus Resurgence in Kitaka Mine Bat Population after Extermination Attempts, Uganda
Source: Emerg Infect Dis. 2014 Oct;20(10):1761–4. doi: 10.3201/eid2010.140696 (PMC4193183; doi:10.3201/eid2010.140696)
Supplement: Technical Appendix — Photographs taken during August 2008–September 2009 of bat extermination efforts at Kitaka Mine, and table showing demographic characteristics of bats captured during a Marburg hemorrhagic fever outbreak investigation at the mine in November 2012, Uganda. [file 14-0696-Techapp-s1.pdf]

Article DOI: http://dx.doi.org/10.3201/eid2010.140696

Marburgviruses in Bats in Mine after Extermination Attempts, Uganda

Technical Appendix

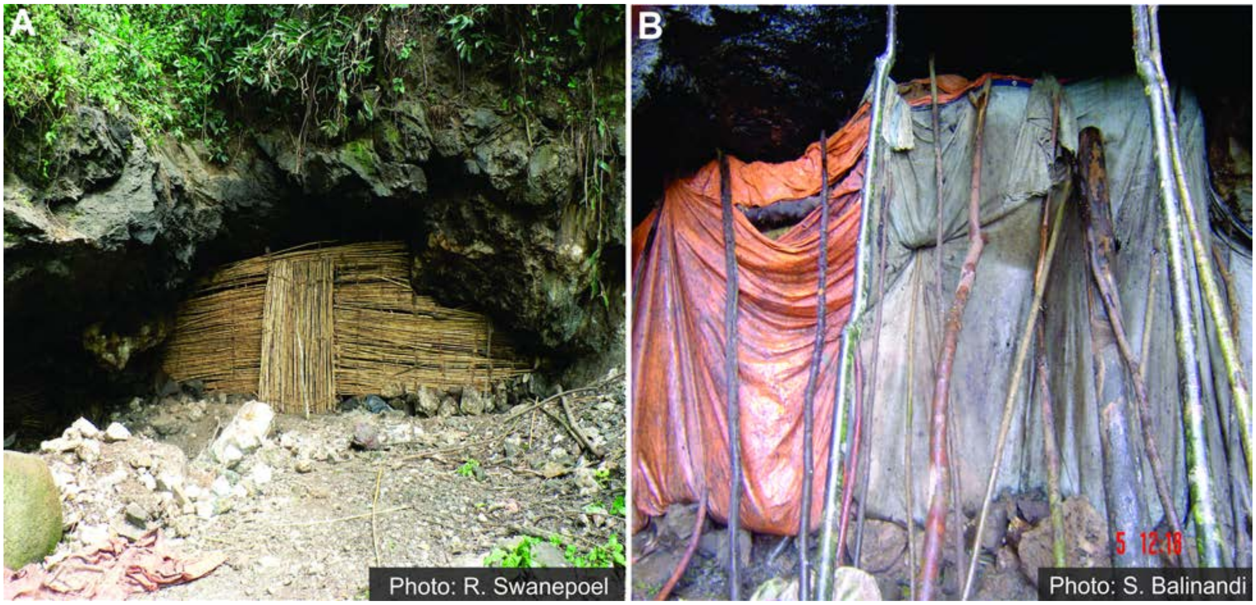

Technical Appendix Figure. Photographs taken at Kitaka Mine, Uganda, between August 2008 and September 2009 showing local efforts to exterminate and exclude *Rousettus aegyptiacus* bats from the mine. A) Papyrus reed walls constructed over entrances and air shafts in the mine. B) Plastic tarps used to completely seal off all access to the mine. Photographs courtesy of R. Swanepoel and S. Balinandi.

Technical Appendix Table. Demographic characteristics of bats captured during a Marburg hemorrhagic fever outbreak investigation at Kitaka Mine, Uganda, November 2012*

		No. (%) marburgvirus-positive bats	
Characteristic	Total no.	Reverse transcription PCR	IgG
Sex			
Female	203	25 (12.3)	30 (14.8)
Male	197	28 (14.2)	36 (18.3)
Age group			
Adult	233	32 (13.7)	46 (19.7)
Juvenile	167	21 (12.6)	20 (12.0)

*All animal work was performed in accordance with protocols approved by the Animal Care and Use Committee, Centers for Disease Control and Prevention.
